# A Method for Evaluating the Green Economic Efficiency of Resource-Based Cities Based on Neural Network Improved DEA Model

**DOI:** 10.1155/2022/9521107

**Published:** 2022-09-08

**Authors:** Zhifeng Shen, Ning Liu, Xialing Li, Zhengguang Kang

**Affiliations:** ^1^Jiangsu University of Technology, Changzhou, Jiangsu 213001, China; ^2^Jiangsu Academy of Science and Technology for Development, Nanjing, Jiangsu 210042, China

## Abstract

In this study, we use BP neural network to improve the DEA model to conduct in-depth research and analysis on the method of green economic efficiency evaluation of resource-based cities. The traditional DEA cannot make ranking and analysis of effective units, which affects the accuracy of empirical analysis. Accordingly, the BP-DEA model is introduced to further conduct a comparative eco-efficiency analysis of relatively effective provinces. In this study, the optimal inputs and outputs are calculated by DEA, and further, the BP neural network is used to fit the functional relationship between the optimal inputs and outputs, and by adding variables, the trained neural network can be used for the prediction of the optimal outputs. In this study, the BP-DEA model is used to empirically investigate the temporal evolution trend, spatial differences, and efficiency differences in eco-efficiency. Meanwhile, breaking through the limitation that DEA can only calculate regional efficiency values, this study combines the Malmquist index to compare and decompose the eco-efficiency of different provinces to analyze the sources of total factor productivity changes. The results show that the method can clarify the gap between the actual operation of each indicator and the reference point; it can identify how much room for improvement still needs to be made for each indicator, and it can also determine whether each city should be rewarded or penalized and its specific amount. Finally, based on the evaluation of eco-efficiency and the main constraints, corresponding policy recommendations are proposed. Finally, based on the evaluation results of the BP-DEA method, this study analyzes the overall efficiency improvement of cities in the two study areas in three dimensions: urbanization construction, ecology, and economic development put forward seven types of urban efficiency improvement and propose targeted urban development suggestions according to regional characteristics.

## 1. Introduction

In the era of big data, how to use big data for effective analysis has become the focus of attention in various industries. Due to the diversity of sources, quantities, structural forms, and other characteristics of big data, it covers many fields, but has a low value density. The existence of data noise and data redundancy in big data sets can have an incalculable negative impact on data analysis. In addition, the functional relationships between multiple variables are also covered in big data sets, which may produce a certain bias in the data analysis results [1]. Therefore, before using big data for analysis and research, data preprocessing should be performed first to eliminate redundant and invalid data. However, traditional big data preprocessing methods do not consider the functional relationships between variables. Data noise and data redundancy in large data sets can have immeasurable negative effects on data analysis. In addition, the large data set also covers the functional relationship between multiple variables, which may cause some deviations in the data analysis results. Therefore, before using big data for analysis and research, it is necessary to preprocess big data to eliminate redundant and invalid data. Data envelopment analysis (DEA) can effectively deal with the bias problem caused by the functional relationship between variables [2]. In the process of data preprocessing by DEA, the most effective data are obtained by filtering the efficiency values, eliminating the outliers and redundant values, and reducing the quantity of data without changing the quality of data. There is no need to predict the functional relationship between input and output variables, and there is no need to set weights in advance, and the most effective data can be obtained by filtering the obtained efficiency values. There is no need to remove outliers and redundant values, and reducing the amount of data without changing the quality of the data is an effective way of data preprocessing that can be applied to machine learning. With urbanization, cities are facing many challenges such as ecological land reduction and environmental pollution, and the contradiction between economic development and the ecological environment is becoming increasingly prominent [3]. Eco-efficiency proposes to maximize value while minimizing resource consumption and environmental pollution, which is an inherent requirement for sustainable urban development. Since the twenty-first century, with the increasing intensity of human activities, the water crisis and ecological deficit have become increasingly serious. At present, the world is facing the common challenge of the water crisis, which not only makes the prospect of economic development a moot point but also may threaten the stability of society and the survival of human beings [4]. In sustainable development, resources and the environment are not only endogenous variables for economic development but also rigid constraints on the scale and speed of economic development. For a long time in the past, governments around the world have focused more on economic growth, placing too much emphasis on GDP growth, and often neglecting issues such as resources and the environment. The problems of resource depletion, environmental degradation, and unsustainability brought about by economic growth have intensified. Clarifying the current situation, spatial differences, and constraints of eco-efficiency have become the primary issue facing sustainable development. Among the existing urban efficiency evaluation methods, DEA has been widely used due to its unique advantages. DEA is a nonparametric technical efficiency analysis method, and it has been more than 40 years since the DEA method was proposed. However, due to the increasing number of elements absorbed by cities in the process of development, they have increasingly evolved into a complex and multilevel system, and with the development of regional integration, the city network system has gradually formed and strengthened, and the competitive effect and complementary effect between cities have emerged. Therefore, it is necessary to improve the relevant urban efficiency evaluation methods. Economic growth has brought about problems such as resource depletion, environmental degradation, and unsustainability, which have intensified. The world is facing the common challenge of water crisis, which will not only make the prospects of economic development impossible but also threaten the stability of society and the survival of human beings.

The way of urban development must shift from focusing on speed and scale in the past to efficiency and quality, i.e., to achieve high-quality urban development [5]. Urban efficiency is a comprehensive reflection of high-quality urban development. At present, reconstructing the urban input-output index system and evaluating urban efficiency are of great significance to promote high-quality urban development with efficiency change. Evaluation of urban efficiency is a hot issue in urban development research, and urban efficiency evaluation has achieved fruitful research results in terms of evaluation methods and evaluation perspectives. DEA is a nonparametric analysis method used to evaluate the relative efficiency of a group of decision-making units (DMUs) with multiple inputs and multiple outputs [6]. With the continuous research on the DEA model, many scholars have made various improvements based on the traditional DEA model. Although the defects such as incomplete ranking and distortion of weights in the traditional model have been compensated, no spatial improvement has been made to the traditional DEA model for cities, which are regional-type evaluation units with spatial attributes. The study of how to improve the DEA model spatially will improve the targeting of urban efficiency evaluation and will be more conducive to improving the rationality of urban resource allocation. Based on the above problems, this study introduces the concept of spatial interaction among cities based on the traditional DEA model, uses the geographic detector tool, combines the modified gravity model, reconstructs the evaluation set in the traditional DEA model by the degree of spatial connection among cities, and realizes the spatial improvement of the DEA model; with the help of BP (back propagation) neural network model, a BP-DEA efficiency evaluation method is proposed.

## 2. Related Works

With the rapid development of information technology, the speed of data analysis and processing has greatly increased, and creating real-time data streams has become a popular trend. To improve their competitiveness, companies need to know not only how to create data quickly but also how to process, analyze, and return it to users quickly to meet their real-time needs [7]. In terms of diversity, the reason for the diversity of data formation is the wide variety of data sources, including various search engines, browsing traces, and social networks; in addition to this, the data are classified in a wide variety of formats. In terms of value, we can conclude that although the value of big data is high, its value density is low; often, only a few hundred or even a few dozen data of thousands of data have value [8]. Big data are not only a revolution of data but also a revolution of thinking, and it has been widely used in government, health care, education, finance, food safety, and other fields, changing our way of life and even our way of thinking. Suocheng et al. [9] used DEA as a tool to preprocess data sets. DEA can identify data that do not match a certain attribute and remove it to get more accurate predictions [9]. Cook et al. [10] demonstrated the high growth rate of DEA-based research. Preprocessing the data based on the DEA method leads to fewer records and less computation, reducing the burden of the study; at the same time, outliers are eliminated to make the subsequent training data more general [10]. The Cobb–Douglas production function model and the Solow economic growth equation are used to calculate the contribution rate of capital, labor, and technological progress to economic growth, and then, the DEA-Malmquist index method is used to calculate the total factor productivity of each province [11]. The goal of removing outliers, preserving universality, and reducing the size of the database used for ANN (artificial neural network) training is achieved. This new data analysis method can improve the prediction accuracy while making the training data set much smaller.

Environment and resources are the general terms for natural factors such as natural existence and a manufactured creation, which lay down the survival, development, and progress of human society; they are characterized by wholeness, scarcity, and value and are the unity of static and dynamic. Resource and environment index system often consists of resource consumption index system and environmental pollution index system [12]. As people pay increased attention to the resources and environment, they gradually realize that the role of resources and environment on economic growth is also becoming increasingly obvious. Peykani et al. [13] analyzed input-output models for 10 sectors to reveal the complex interrelationship between energy, environment, and economic welfare, proposed pollution emission factors and European sulfur deposition carriers, and studied the relationship between energy use, and environmental impact, employment, and economic development [13] Kononets et al. [14] analyzed and evaluated the environmental carrying capacity of Anhui Province by establishing the entropy TOPSIS model and came up with four key factors affecting the environmental carrying capacity of Anhui Province [14]. Tan et al. [15] argued that the current research on the bearing capacity of resources and environment has not yet formed a unified systematic theory, and should strengthen the basic theory and bearing mechanism, technical standards and norms, evaluation, and system integration research so that the research on the bearing capacity of resources and environment can be more standardized and systematic for the better benefit of human beings [15].

In terms of research on the application area of eco-efficiency, foreign countries have more research on enterprises and product systems. The research organically combines factors such as product development and design, system structure, and identification of key issues with eco-efficiency research and applies the results to the process of healthy and sustainable development of enterprises. China is more inclined toward regional studies, such as the Western provinces, six central provinces, Beijing-Tianjin-Hebei city cluster, and the middle reaches of the Yangtze River city cluster. By comparing the differences in eco-efficiency among cities, targeted improvement strategies are proposed in terms of policy, management, and technology. Secondly, in terms of evaluation methods, the single ratio analysis method, indicator system analysis method, and model analysis method described above have their advantages and disadvantages, and appropriate evaluation methods need to be selected according to the purpose of the study and the characteristics of the research object. With the advancement of urbanization, cities are faced with many challenges such as the reduction in ecological land and environmental pollution, and the contradiction between economic development and ecological environment has become increasingly prominent. Overseas research on the theory and application of model analysis and single ratio analysis has made great progress. In terms of the use of the DEA model, foreign studies have improved the traditional DEA model to different degrees and aspects based on the characteristics of the evaluation unit [16]. Related applied studies have shown an increasing trend in recent years. However, since most of the studies are based on the application of existing models, scholars have not further explored and compared the principles, applicability, and accuracy of the models, resulting in certain discrepancies in the results of eco-efficiency assessment, which can directly influence the government's decision-making and thus may lead to further deterioration of resources and environment. Based on this, this study discusses the characteristics of the base model of DEA and improves the DEA model with the help of the BP neural network model to meet the research of eco-efficiency in this study.

## 3. Methodology

### 3.1. Data Envelope Analysis

Charnes first proposed data envelopment analysis (DEA) in 1978, in which a set of decision-making units (DMUs) of the same type is selected, and the relative efficiency of the DMUs is calculated by analyzing data on input and output indicators through a mathematical planning model and then determining the relative effectiveness of the DMUs and whether scale efficiency and technical efficiency are achieved. DEA is a nonparametric statistical method that can obtain DEA models from input and output data and perform economic analysis. Therefore, DEA is highly valued by the international academic community and has become the most common method in the field of efficiency evaluation. For a given complex large data set, even if there are many kinds of variables in the data set and the relationship between variables is complicated, DEA preprocessing can perfectly avoid the negative factors with its own unique mathematical planning advantages and complete the efficiency evaluation of variable indexes. When evaluating the efficiency of DMU based on the DEA data preprocessing method, it is not necessary to know the attribute relationships embedded inside the complex data set in advance, and it is not necessary to determine the variable weights to obtain the effective DMU and provide the improvement direction and adjustment values for the invalid DMU, which has certain advantages in this study.

BP neural network is a multilayer feedback network trained by the error back propagation algorithm proposed by a group of scientists led by Rumelhart and McCelland in 1986 as an artificial intelligence information processing system capable of learning nonlinear functional relationships between variables and underlying patterns. This neural network model is characterized by the fact that it does not need to assume the mapping relationship between the input and output in advance, but uses the learning adaptation ability among the network nodes to map the fitting result with the minimum distance from the desired output value through continuous training [17]. Therefore, it is more common in the research of artificial neural network model applications. The whole computational process of this BP algorithm includes the positive propagation of the signal and the inverse propagation of the error. That is, the error output is from the output point to the input point, while the adjustment weights and thresholds are from the input point to the output point. In the positive propagation process, the input signal is transmitted to the implicit layer through each node of the input layer and then transmitted to the output layer through the nonlinear processing of the implicit layer to complete the positive transmission of the signal. If the actual output does not match the expected output, the back propagation of the output error occurs. During the back propagation, the output error is transferred to the upper layer, and the weights and thresholds of that layer are corrected in the direction of gradient descent [18]. The network training keeps reducing the error along the gradient direction until the termination condition is met, and the termination condition is usually set to the minimum error or the maximum number of training sessions to obtain the weights and thresholds under that training expectation. In this study, we take a common single hidden layer BP neural network as an example to illustrate the self-learning process of BP networks, i.e., the BP algorithm. By correcting the error and adjusting the connection weights and thresholds, the training is repeated until the error is less than the set minimum error, and then, the learning process is finished, and the trained network can map the functional relationship between the variables and the underlying patterns more accurately, to realize the prediction function of the network.

(1) $$\begin{array}{c} Q_{j}=f\left(\underset{i,j=1}{\sum }W_{ij}\right)\times f\left(x_{i}-q_{j}\right),\\ Y_{i}=f\left(\underset{j,k=1}{\sum }T_{jk}\right)-f\left(O_{j}\times q_{k}\right)\text{.} \end{array}$$

Among them, *f* is the action function; *q* is the neuron threshold.

DEA is an efficiency measurement method based on linear programming ideas with a strict mathematical derivation process, so the model can provide target values and improvement amounts for invalid decision units, but this also leads to certain limitations in the application of the method; i.e., it is more sensitive to evaluation index data and only suitable for evaluating decision units with objective values (nonpredicted values) and lacks certain predictive ability. In contrast, BP neural network as an artificial intelligence information processing system can learn the nonlinear functional relationships between variables and the underlying patterns, and its advantage lies in the predictive capability. The general idea of modeling in this study is to use the input-output index values from 2015 to 2021 as the BP neural network input and the comprehensive efficiency values measured by the CCR model as the BP neural network output, in which a total of 70 samples (70%) of the Yangtze River Economic Zone data from 2015 to 2018 are selected as the network training samples, and a total of 30 samples (30%) from 2019 to 2021 are selected as the test samples of the network. Through the learning and training of the BP neural network, the underlying relationship between the input-output index and the comprehensive efficiency is fitted, and when the trained efficiency prediction model passes the test, it shows that the model has a strong generalization ability.

According to the definition of the efficiency evaluation index, the efficiency evaluation of a province can be seen as the integration of all the input and output indicators into a relatively representative index, and then, the ratio of a single input to a single output is calculated. Assuming that the calculated province is denoted as DMU_*jo*_(*jo*=1,2, ..., m), the optimal weight vectors *q* and *p* are chosen to satisfy the maximum value of the evaluation index *k*_*jo*_ for the province, if none of the provincial efficiency indices is greater than 1. The evaluation index *k*_*jo*_ for a province refers to the eco-efficiency value of the*j*_0_ evaluated provincial area, which is the following 0 < *k*_*jo*_ < 1. Its economic significance is the extent to which the input index of the evaluated *j*_0_ can be scaled down if the economic output of the evaluated can be replaced by any other linear combination of regional outputs, and the magnitude of the scaling down ratio is *k*_*jo*_. If the eco-efficiency of the region is valid, *k*_*j*_0__=1; when *k*_*jo*_ < 1, 1 − *k*_*jo*_ represents the maximum proportion of input reduction. DEA linear programming model is mainly used to discriminate whether the output of each province is valid or not, and the model to discriminate the validity of the input of the study object (*L*^·^) is obtained by doing the Charnes–Cooper transformation on the inverse form of the objective function of the model ($\overset{-}{L}$).

(2) $$\begin{array}{c} k_{jo}=\min\frac{p^{t}y_{jo}}{q^{T}x_{jo}},\\ s.t\left\{\begin{array}{c} \varpi^{T}+q^{T}y_{jo}\leq 0\\ \varpi \geq 0\\ \left(j=1,2,...,n\right) \end{array}\right.\text{.} \end{array}$$

### 3.2. BP-DEA Model Structure Design

**Figure 1:**
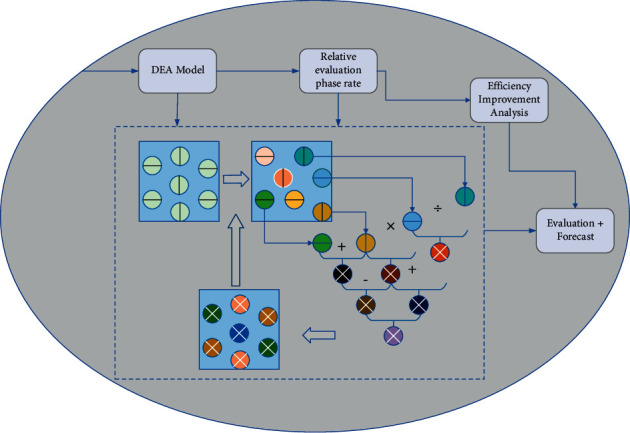
BP-DEA network comprehensive evaluation model.

The DEA method has its advantages in dealing with multiple input and multiple output problems, which largely avoids the influence of human subjective factors, and its evaluation results are more scientific and accurate. However, the DEA method also has its defects, which cannot be further simulated and predicted and cannot provide clear solution ideas for decision-makers. Since BP neural network requires training samples and corresponding tutor values, this process still cannot avoid the need to use other methods to assist in the evaluation, and the more common is the use of the expert scoring AHP method. This inevitably introduces the human subjective factor, and the evaluation period will be prolonged by the time of collecting expert experience scores [19]. Because of the respective advantages and shortcomings of the above two methods, this study organically combines the two methods for science and technology achievement transformation evaluation. Firstly, the original evaluation index data are input into the DEA model to calculate the comprehensive evaluation value of the transformation of scientific and technological achievements based on the DEA model, and then, the BP neural network is trained with this evaluation value as the tutor value. The BP-DEA neural network comprehensive evaluation model constructed in this study is shown in Figure 1. On the other hand, its relative efficiency evaluation value can be used as a piece of objective tutor information to replace the subjective expert rating used in the traditional BP network, to guide the BP. On the other hand, its relative efficiency evaluation value can be used as objective tutor information to replace the subjective expert rating used in traditional BP networks, thus guiding the BP neural network to self-learning and training, and forming a stable network structure for specific evaluation and prediction.

To make the information provided by the DEA method more accurate and scientific, it is necessary to first clarify the objective of the evaluation, analyze the decision unit around this objective, including the main objective, subobjectives, and factors affecting these objectives, and establish a hierarchical structure. Then, the nature of various factors is clarified, distinguishing whether the factors are variable, controllable, and primary and secondary relationships. Then, the quantitative and qualitative relationships among the factors are considered. Sometimes, it is also necessary to distinguish whether the decision unit has boundaries or not and to clarify the structure and hierarchy of the decision unit [20]. When the evaluation problem is determined, it is necessary to determine the indicators that can reflect the evaluation objectives and reflect the qualitative relationships among the indicators into the weight constraints, to construct the evaluation indicator system. Then, the decision unit is selected; i.e., the reference set is determined, and the selected reference set must have the same objectives, tasks, external environment, and input-output indicators, and the decision unit should be representative to a certain extent. Then, the index data are collected and organized, and finally, the appropriate DEA model is selected for calculation and analysis according to the purpose of validity analysis and the actual problem context. According to the calculation results obtained in Stage 2, we analyze and compare them, find out the reasons for invalid units, and give improvement suggestions.

**Figure 2:**
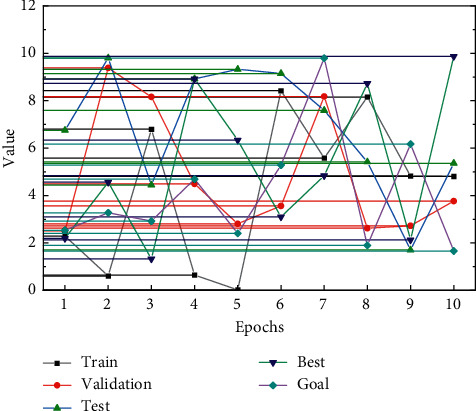
Relationship between the number of neurons in the hidden layer and the number of trainings.

The BCC model is obtained by widening the premise assumption of constant payoffs of scale in the CCR model by adding a condition ∑_*j*=0_*λ*_*j*_=1 to obtain the BCC model under variable payoffs of scale. Since the model considers different forms of payoffs to scale, the efficiency measured by the BCC model is purely technical. In case of negative values of input-output indicators, the BCC model can be used to process a linear transformation of the data. In addition to the three formulas that can be consulted to roughly determine the number of neurons in the hidden layer, there is another method that can be used to determine the number of neurons in the hidden layer. The first step is to make the number of implicit neurons variable or to put in enough implicit neurons to eliminate those that do not work by learning until they are not contractible. Similarly, it is possible to start with a relatively small number of neurons, learn a certain number of times, and then increase the number of hidden layer neurons if unsuccessful, until a reasonable number of hidden units is reached. These two methods are combined to determine the number of hidden units [21]. First, the reference formula shows that the approximate number of hidden units is around 5. For this reason, it is possible to set the loop and check when the network is more efficient and the output error is smaller when the number of hidden units is taken between 4 and 6. The errors and effects during training are compared to determine a reasonable number of hidden units. As shown in Figure 2, when the hidden layer neurons are in 4–6, both train twice to reach the target error, but the error message is different for different hidden layer neurons. When the hidden layer neurons are 4, the validation set error is 0.0034; when the hidden layer neurons are 5, the validation set error is 0.0087; and when the hidden layer neurons are 6, the validation set error is 0.0064, so the number of hidden layer neurons is 4.

## 4. Design of Evaluation Indicators for Green Economic Efficiency of Resource City-Type Cities

System theory is to treat the specific object under study as an overall system and sorts out the interactions among the constituent elements of the system with the basic idea of starting from a holistic perspective and grasping its internal structure and dynamic changes to achieve the goal of optimizing the whole. The basic attributes of the system include wholeness, hierarchy, correlation, dynamics, and purpose. In constructing the theoretical analysis framework of the factors influencing the efficiency of construction land use, not all factors act independently on the efficiency of construction land use, but directly or indirectly influence the efficiency of construction land use through interconnected pathways; i.e., most of the influencing factors have a strong correlation with each other, and all, in other words, most of the influencing factors have a strong correlation with each other, and all the influencing factors can be regarded as a whole system.

From 2003 to 2005, with the rapid development of industrialization and urbanization, energy consumption intensity and major pollutant emissions showed an upward trend. The energy consumption per unit of GDP rose by 9.8%, and the total emissions of sulfur dioxide and chemical demand increased, respectively, by 32.3% and 3.5%. At the same time, these few years are an important time period for the accumulation of capital investment. During this period, a large amount of capital flows to the secondary industry, which leads to the ineffectiveness of the scale and management of this industry to a certain extent. At the same time, because of not giving up the use of the original backward mechanical equipment, there is even a regression in technology. In terms of environmental governance and protection, the discharge of many environmental pollutants has not reached the predetermined target. During this period, China was highly dependent on coal energy, but due to backward equipment, the utilization efficiency of coal resources was relatively low, and many equipment using coal resources did not have “desulfurization” devices, which made the pollutant emissions in some areas very high serious.

From 2006 to 2010, the significant improvement of regional total factor energy efficiency was mainly due to the reversal of the rising trend of energy consumption intensity and major pollutant emissions during the period of the government's “Eleventh Five-Year Plan” during the rapid development stage of industrialization and urbanization. During the “Eleventh Five-Year Plan” period, the energy consumption per unit of GDP has changed from an increase of 9.8% in the three years after the “Tenth Five-Year Plan” to a decrease of 19.1%. The increase of 32.3% and 3.5% turned into a decrease of 14.29% and 12.45%, and the energy efficiency level and environmental quality were significantly improved. At the same time, the adjustment of industrial structure and the further improvement of infrastructure have also promoted the improvement of total factor energy efficiency to a certain extent.

**Figure 3:**
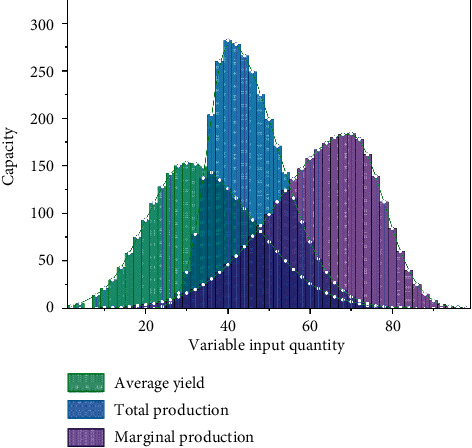
Variable factor inputs versus yield.

The law of diminishing marginal efficiency, also known as the law of diminishing marginal utility, is one of the basic principles in the field of economics [22]. Increasing the input of this resource may lead to a decrease in the output volume, i.e., the output volume of the increased unit resource is decreasing, as shown in Figure 3. When the input of construction land is too small compared with the input of other factors, increasing the input of construction land will not only lead to an increase in total output but also lead to an increase in the marginal output of a new unit of construction land, but once the appropriate ratio between factors is crossed, increasing the input of construction land makes the increase in total output. However, once the ratio between factors is exceeded, the increase in total output is not obvious and the marginal output per unit of new construction land decreases gradually.

To address the shortcomings of this study, this study incorporates natural ecosystems into the evaluation of urban eco-efficiency and selects ecosystem service values and landscape diversity indicators as the characterization indicators of natural ecosystems, taking 34 prefecture-level cities in *X* Province as examples for the specific measurement of ecosystem indicators. Natural ecosystems provide the material basis for human survival and development. Humans can obtain the food, energy, and water they need from natural ecosystems, and they can also obtain various benefits such as disaster prevention and carbon sequestration, and rest, all of which reflect the welfare that nature gives to human survival development. Therefore, the value of final products and services provided by natural ecosystems to human beings should also be used as an important indicator to measure the sustainable development of a country. Based on the above analysis, this study incorporates indicators characterizing natural ecosystems into urban eco-efficiency assessment and selects the value of ecosystem services and landscape diversity indicators as indicators characterizing natural ecosystems [23]. The value of ecosystem services and the landscape diversity index characterize the value and structure of natural ecosystems, and it is difficult for social statistics to show their changes. Due to the specialized nature of data acquisition and data processing, these indicators are rarely used in the social science field, and it is difficult to obtain data on ecosystem structure indicators. Natural ecosystem data usually require remote sensing and GIS spatial technology to identify the changes in ecosystem structure and reflect the differences between regions. In this study, we use ESA global land cover data for 34 cities in *X* Province, select 2012, 2015, 2018, and 2021 as the study years with a 3-year interval, and use GIS technology to spatially process the data to measure the natural ecosystem index data of 34 cities in the region during 2012–2021, to further use the model to measure regional ecosystem indicators. The data were spatially processed using GIS technology to measure the natural ecosystem indicators of 34 cities in the region during 2012–2021.

In this study, we firstly processed the acquired spatial data, and the main processing method was ArcGIS spatial technology method. First, based on the national resource and environmental database classification standard and land cover remote sensing classification system, combined with the ecological environment of *X* Province and other information, we identified and classified the ecosystem types in Northeast China. Since ecosystem services involve many aspects, and the connection between ecological processes and economic values is complex, there are still many uncertainties in the understanding of ecosystems, and there is a certain roughness in the value assessment of ecosystems. In the actual measurement, the formula for calculating the value of ecosystem services is as follows. Based on the diverse characteristics of big data sources, quantities, different structural forms, and real-time nature, the value it covers is very high, but its value density is very low. Data noise and data redundancy in large data sets can have immeasurable negative effects on data analysis.

(3) $$\begin{array}{c} ESV=\sum_{k=1}^{n}A_{k}\times VC_{k.} \end{array}$$

The total value of ecosystem services ($) in the study area is represented by *k*, the number of ecosystem types, *VC*_*k*_ is the coefficient of total ecological service value per unit area of ecosystem type *k* ($/hm^2^), and *A*_*k*_ is the area of ecosystem type *k* in the study area (hm^2^).

In recent years, the application of diverse tools such as remote sensing and geographic information system (GIS) in landscape ecology has enabled the quantitative measurement of landscape diversity. The SHDI can characterize the changes in landscape structure and function over time and reflect the complexity of the landscape. The SHDI usually takes the value range of SHDI ≥ 0, and the larger the SHDI is, the more complex the landscape is. The SHDI index can not only calculate and analyze the changes in diversity and heterogeneity of different landscapes in the same period but also compare the changes in the same landscape in different periods.

## 5. Analysis of Results

### 5.1. BP-DEA Model Results

Put in a relatively small number of neurons at the beginning, after learning a certain number of times, if unsuccessful, the number of neurons in the hidden layer is increased until a reasonable number of hidden units are reached. In the evaluation results of traditional standard DEA, there are often cases where the efficiency of multiple DMUs is all 1, and both more DMUs are relatively efficient, and at this time, the standard model cannot distinguish these DMUs that are also efficient. In the radial DEA model, the measurement of the degree of inefficiency only includes the proportion of all inputs and outputs that are reduced or increased in equal proportion. For the inefficient DMU, the gap between its current state and the strongly efficient target value includes a slack improvement component in addition to the equiproportional improvement component, which is not captured in the efficiency value measurement.

To avoid singular data, which in turn affects the analysis results, this study needs to adopt normalization operations for the sample data. When each index component of the input/output vectors of the model does not have the same dimension, each index component needs to be normalized in the respective value domain [24]. When each index component of the input/output vectors has the same magnitude and the same size, it needs to be normalized in the whole sample data value domain. Considering the output range of the BP neural network transfer function and the type of each evaluation index, the maximum-minimum normalization method is chosen to process the sample data in this study.

(4) $$\begin{array}{c} X^{*}=\frac{x-\min\;A}{\max\;A+\min\;A}\text{,} \end{array}$$

where *x* is a raw value of attribute *A*, min *A* and max *A* are the minimum and maximum values of attribute *A*, respectively, and *X*^*∗*^ is the standard value after the maximum-minimum normalization method, and the range is 0 ≤ *X*^*∗*^ ≤ 1.

**Figure 4:**
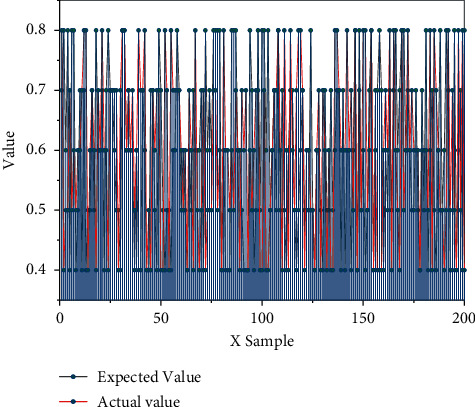
Comparison of the true and predicted values of the training set.

BP Neural Network Training: where the number of training steps is set to 1000, the initial learning rate is 0.05, the error value at the end of training is 0.001, and the learning rate is 0.05. When the training is completed, the training results need to be checked, and the error between the actual output value of the training set and the desired output value is analyzed to see whether the error between the two meets the accuracy requirements. If the accuracy requirements are met, it means that the training of the neural network has been completed and the test set can be tested, and if the accuracy requirements are not met, it is responsible for the need to adjust the parameters and retrain. The maximum relative error in the training sample is 3.43%, which is less than 5% of the accuracy requirement, indicating that the training is qualified and the BP neural network model has been trained. The results of comparing the real value of the training set with the predicted value are shown in Figure 4. The way of urban development must shift from focusing on speed and scale in the past to efficiency and quality, that is, to achieve high-quality urban development.

As can be seen from Figure 4, the expected output of the BP network model prediction sample is consistent with the actual output, indicating that the network model can better reflect the mapping relationship between the input and output values, and we can evaluate the efficiency of the terminal through this model. Using the AHP-based BP neural network evaluation model to evaluate the operation condition of *X* Province and analyze the current efficiency level of *X* Province is important for the enterprises in *X* Province to make and implement a series of targeted development strategies based on the actual operation condition, and then, the current operating situation of the enterprise is analyzed and judged whether the enterprise needs to reallocate the existing resources based on the results of the efficiency evaluation, to enhance the efficiency of the enterprise and improve the operational efficiency. In this stage, the most important step is to conduct an efficiency analysis, explore the factors affecting the efficiency of the enterprise, analyze the reasons affecting the efficiency of the enterprise in *X* Province, and then give corresponding improvement suggestions based on the specific reasons to improve the efficiency of the enterprise and increase the core competitiveness of the enterprise. If the input and output indicators have negative values, the BCC model can be used to linearly transform the data.

**Figure 5:**
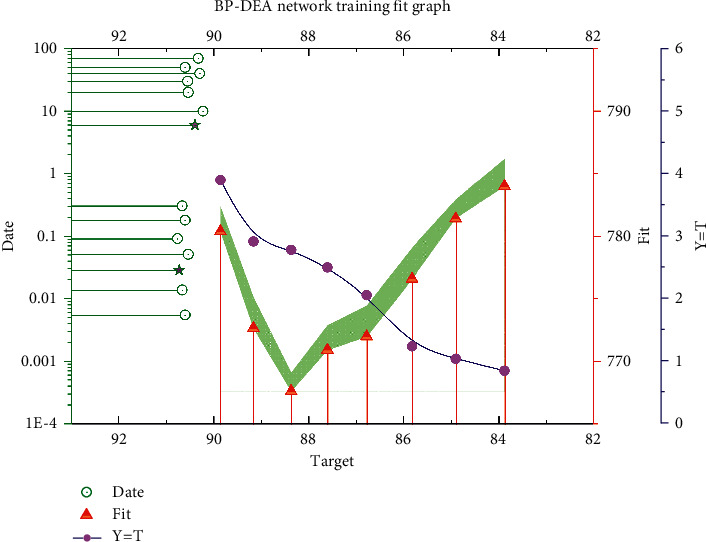
BP-DEA network training fit graph.

The learning rate is related to the speed of weight change at each step of training, and if the weights of the model change quickly, it will cause high-frequency oscillation of the loss function, and if the weights change too slowly, the harder the model will be to converge, and then, it will take longer to learn the training, but usually, the smaller the learning rate, the better the model learns, and the better it is for BP neural network training to approach the minimum. Therefore, a smaller value is preferred for tuning, so a learning rate in the range of [0.01, 0.08] is usually tried. By comparing the number of training steps and errors in the table, it can be found that the model performance is optimal when taking 0.01, so the net learning rate of the efficiency prediction model is determined to be 0.01. The network training results are shown in Figure 5.

### 5.2. Simulation Experiment of Green Economic Efficiency Evaluation Model for Resource City-Type Cities

**Figure 6:**
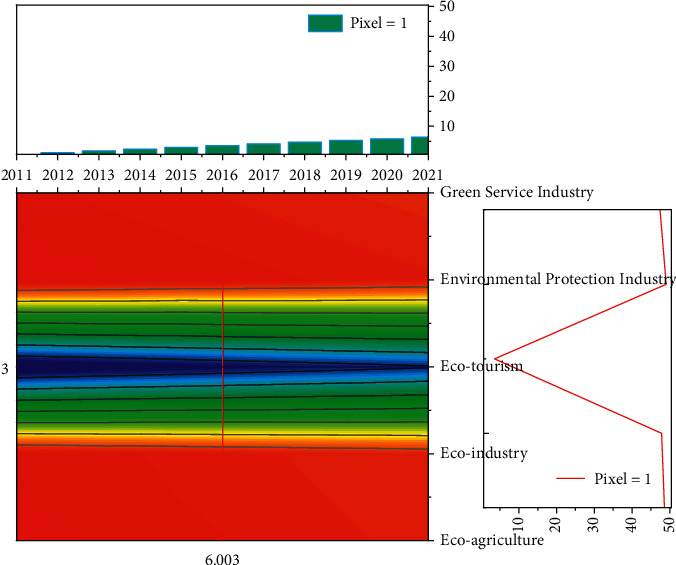
2011–2021 X Province turnover trends.

To explore the efficiency situation of the economic belt of Province *X* in 2022, and to better provide efficiency improvement suggestions for the relevant sector managers, a forecast-based scenario is set in this study. By forecasting the turnover of Province *X* in 2022, the efficiency prediction model constructed in the previous paper is used to measure the efficiency of Province X in 2022 under the projected road transport output when matching the current 2018 inputs, to provide a reference benchmark for the relevant managers to improve efficiency. As can be seen from Figure 6, the trend of turnover in Province X over time from 2010 to2018 is in a nonlinear form, and this study only forecasts for the next two periods, so it is suitable to use the three-time exponential smoothing method, and the Excel tool is selected to build the forecasting model. The termination condition of the BP algorithm is usually set as the minimum error or the maximum training times, to obtain the weights and thresholds under the training expectations.

**Figure 7:**
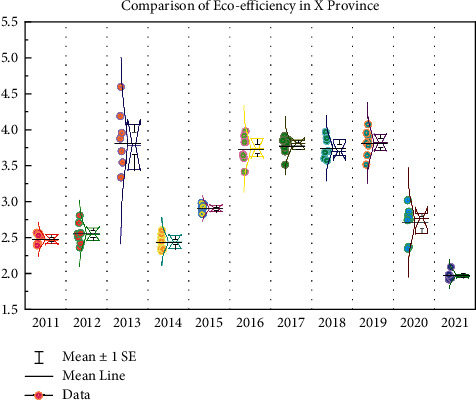
Comparison of eco-efficiency in X Province.

After the parameters are estimated by the SFA regression model in the second stage, the provinces are adjusted to the same external environment and random error scenario so that each province faces the same business environment and conditions, thus further adjusting the resource environment input variables in the first stage to exclude the external environment factors, and finally substituting the adjusted input variable values with the output values in the first stage into the data envelope model again for the count. Finally, the adjusted input variable values and the output values of the first stage were again substituted into the data envelope model for calculation. Overall, the overall level of adjusted eco-efficiency in China is low, with large internal differences, and the trend of change in the east, middle, and west has also changed, showing approximately an “M”-shaped trend, with two transitions, in 2007 and 2013, respectively. As shown in Figure 7, after excluding external environmental variables and random disturbances, the regional eco-efficiency of each province has changed significantly. Compared with the first stage, the eco-efficiency has decreased significantly, by about 11.6%, which indicates that the external environmental factors and random luck disturbances that cannot be controlled can overestimate the eco-efficiency level of each province region to a certain extent.

In essence, eco-efficiency contains both resource-saving and environment-friendly contents. With the rapid development of the economy, a series of problems such as resource scarcity and environmental pollution have gradually become prominent, which restrict the sustainable development of the economy. Although in recent years, energy has become increasingly scarce and environmental pollution has become more serious, a series of corresponding measures taken by our government has made our overall eco-efficiency still in a slow upward trend, which of course is inseparable from our scientific and technological progress, the improvement of people's awareness of environmental protection, and the improvement of people's cultural literacy. A DMU of the same type refers to a set of DMUs with the same tasks and goals, the same environmental conditions, and the same input and output metrics.

## 6. Conclusion

This study builds a regional ecological efficiency evaluation model based on BP neural network improved DEA model and evaluates the economic performance under the constraints of resources and environment. In this study, the use of DEA can effectively deal with the problem of deviation caused by the functional relationship between variables. The amount of data without changing the data quality is reduced, and the BP-DEA model is used to study and analyze the resource utilization and its influencing factors. The optimal input and output are calculated through DEA, and the BP neural network is further used to fit the functional relationship between the optimal input and output. By adding variables, the trained neural network can be used to predict the optimal output. Combined with the Malmquist index, the ecological efficiency of different provinces is compared and analyzed, and the gap between the actual operation of each indicator and the reference point is clarified. This research has achieved certain achievements in the evaluation method of green economic efficiency of resource-based cities. However, limited by personal knowledge ability and practical conditions, the study still has some deficiencies. The result measured by the BP-DEA method is relative efficiency, and there are still limitations in the analysis of time series for a certain decision-making unit. The parameter setting of BP neural network is relatively cumbersome, and there is no more direct selection method. Later, the heuristic algorithm can be introduced to simplify the setting process of relevant parameters.

## Data Availability

The data used to support the findings of this study are included within the article.

## References

[1] Jin Z. (2021). Retracted article: green city economic efficiency based on cloud computing and machine learning. *Arabian Journal of Geosciences*.

[2] Cui K., Jing X. (2019). Research on prediction model of geotechnical parameters based on BP neural network. *Neural Computing & Applications*.

[3] Zhang Y. G., Tang J., Liao R. P., Zhang M. F.Y., Wang X. M. Y. (2021). Application of an enhanced BP neural network model with water cycle algorithm on landslide prediction. *Stochastic Environmental Research and Risk Assessment*.

[4] Deng Y., Xiao H., Xu J., Wang H. (2019). Prediction model of PSO-BP neural network on coliform amount in special food. *Saudi Journal of Biological Sciences*.

[5] Zhang L., Wang F., Xu B., Chi W., Wang Q., Sun T. (2018). Prediction of stock prices based on LM-BP neural network and the estimation of overfitting point by RDCI. *Neural Computing & Applications*.

[6] Ding S., Su C., Yu J. (2011). An optimizing BP neural network algorithm based on genetic algorithm. *Artificial Intelligence Review*.

[7] Sadeghi B. H. M. (2000). A BP-neural network predictor model for plastic injection molding process. *Journal of Materials Processing Technology*.

[8] Wei Q., Zhang J., Zhang X. (2000). An inverse DEA model for inputs/outputs estimate. *European Journal of Operational Research*.

[9] Suocheng D., Zehong L., Bin L., Mei X. (2007). Problems and strategies of industrial transformation of China’s resource-based cities. *China Population, Resources and Environment*.

[10] Cook W. D., Liang L., Zha Y., Zhu J. (2009). A modified super-efficiency DEA model for infeasibility. *Journal of the Operational Research Society*.

[11] Ganji S. S., Rassafi A. A. (2019). DEA Malmquist productivity index based on a double-frontier slacks-based model: Iranian road safety assessment. *European Transport Research Review*.

[12] Seyedalizadeh Ganji S. R., Rassafi A. A. (2019). Measuring the road safety performance of Iranian provinces: a double-frontier DEA model and evidential reasoning approach. *International Journal of Injury Control and Safety Promotion*.

[13] Peykani P., Mohammadi E., Seyed Esmaeili F. S. (2019). Stock evaluation under mixed uncertainties using robust DEA model. *Journal of Quality Engineering and Production Optimization*.

[14] Kononets N., Ilchenko O., Mokliak V. (2020). Future teachers resource-based learning system: experience of higher education institutions in Poltava city, Ukraine. *The Turkish Online Journal of Distance Education*.

[15] Tan M., Zhao H., Li G., Qu J. (2020). Assessment of potentially toxic pollutants and urban livability in a typical resource-based city, China. *Environmental Science and Pollution Research*.

[16] Qin Y., Luo Y., Lu J., Yin L., Yu X. (2018). Simulation analysis of resource-based city development based on system dynamics: a case study of Panzhihua. *Applied Mathematics and Nonlinear Sciences*.

[17] Wang L., Wang Y., Sun Y., Han K., Chen Y. (2022). Financial inclusion and green economic efficiency: evidence from China. *Journal of Environmental Planning and Management*.

[18] Zhang M., Li B. (2022). How to design regional characteristics to improve green economic efficiency: a fuzzy-set qualitative comparative analysis approach. *Environmental Science and Pollution Research*.

[19] Naseer S., Song H., Aslam M. S., Abdul D., Tanveer A. (2022). Assessment of green economic efficiency in China using analytical hierarchical process (AHP). *Soft Computing*.

[20] Wang Z., Wang X., Liang L. (2019). Green economic efficiency in the Yangtze River Delta: spatiotemporal evolution and influencing factors. *Ecosystem Health and Sustainability*.

[21] Li Q. (2019). Regional technological innovation and green economic efficiency based on DEA model and fuzzy evaluation. *Journal of Intelligent and Fuzzy Systems*.

[22] Zheng X., Yu H., Yang L. (2022). Technology imports, independent innovation, and China’s green economic efficiency: an analysis based on spatial and mediating effect. *Environmental Science and Pollution Research*.

[23] Zeng G., Geng C., Guo H. (2020). Spatial s effect of strategic Emerging Industry agglomeration and green Economic Efficiency in China. *Polish Journal of Environmental Studies*.

[24] Ganji S. S., Rassafi A. A. (2019). Road safety evaluation using a novel cross efficiency method based on double frontiers DEA and evidential reasoning approach. *KSCE Journal of Civil Engineering*.

